# Depression Is Associated With the Absence of Sex Differences in the 2D:4D Ratio of the Right Hand

**DOI:** 10.3389/fpsyt.2019.00483

**Published:** 2019-07-16

**Authors:** Simon Sanwald, Katharina Widenhorn-Müller, Jennifer Wernicke, Cornelia Sindermann, Markus Kiefer, Christian Montag

**Affiliations:** ^1^Department of Psychiatry, Ulm University, Ulm, Germany; ^2^Department of Molecular Psychology, Institute of Psychology and Education, Ulm University, Ulm, Germany

**Keywords:** prenatal testosterone, 2D:4D ratio, case–control, depression severity, major depression, sex, gender role

## Abstract

The 2D:4D digit ratio reflects prenatal testosterone relative to estradiol exposure of a developing embryo. Higher levels of prenatal testosterone have been related to lower 2D:4D ratios. In addition, higher 2D:4D ratios have been associated with female gender, neuroticism, and depression severity. Therefore, the present study investigated whether 2D:4D ratios differ between inpatients with major depression and matched healthy controls and whether 2D:4D ratios correlate with depression severity. We examined 139 inpatients diagnosed with major depression according to the Diagnostic and Statistical Manual of Mental Disorders (DSM-IV) criteria and 137 healthy controls regarding 2D:4D ratios of both hands and BDI-II scores. While we observed significant sex differences in the 2D:4D ratio of the right hand in the healthy control group (women on average showed a significantly higher 2D:4D ratio), no such differences were found in the group of depressed patients. The 2D:4D digit ratios did not correlate with depression severity even when examined for group and sex separately. We conclude that major depression is associated with an absence of sex differences in the 2D:4D ratio.

## Introduction

Major depression is a burden for affected individuals, their families, and the society due to costs for treatment, hospitalization, and loss of productivity (1). Depressive disorders are characterized by prolonged or permanent feelings of sadness, lack of interest, hopelessness, and exhaustion (2). This mirrors also in lower SEEKING and higher FEAR/SADNESS, hence primary emotional systems according to affective neuroscience theory (3) [ANT; for a recent introduction into ANT, please see Ref. (4)]. Women have a two-fold higher risk to develop episodes of depression than do men and in addition experience more severe episodes than men (5). It has been suggested that the observed sex-related differences in the prevalence of psychiatric disorder might be associated with the exposure of the embryo to testosterone, the male sex hormone, during development (6). The ratio of the index finger (2D) relative to the ring finger (4D) has been found to be associated with androgen-to-estrogen signaling during digit development in an animal model. Digit 4 was affected more strongly than digit 2 by both androgen receptor and estrogen receptor α activity. Inactivation of the androgen receptor decreased growth of digit 4 resulting in a higher 2D:4D ratio. In contrast, inactivation of estrogen receptor α increased growth of digit 4, which led to a lower 2D:4D ratio (7). To date, there are six studies investigating the associations between prenatal/perinatal androgen/estrogen concentrations and 2D:4D ratios in humans. Three studies found an association (8–10), while three studies did not find a significant association (11–13). Lower 2D:4D ratios have previously been shown to be associated with male sex (14). Sex differences in 2D:4D ratios have been found to be more pronounced in the right hand (14, 15). Exposure to higher levels of prenatal testosterone is associated with the development of stereotypically male personality traits (16) coinciding with findings showing an association of lower 2D:4D ratios and aggressive tendencies (17) as well as addictive tendencies towards gaming disorder (18, 19). A study by Montag and colleagues (20) highlighted that female persons who stutter suffer more from stuttering when they had more male hands. On the other hand, a higher, more feminine, 2D:4D ratio has been associated with schizotypal personality traits (21) and neuroticism in women (22, 23).

However, only a small number of studies with heterogeneous results examined whether a non-clinical population with 2D:4D finger length is associated with depressive symptoms (the latter also being linked to trait neuroticism) (24, 25). In a sample of 298 psychology students, Bailey and Hurd found that males with higher, more feminine 2D:4D ratios have higher depression scores. This association was only observed for the digit ratio of the right hand (26). Martin and coworkers (27) investigated the finger length ratio and depression severity in a sample of 52 men and 50 women. The authors observed a non-significant negative correlation between 4D digit length and Beck Depression Inventory (BDI) scores in men and suggested that high prenatal testosterone might predispose men to depression.

In a study with 128 undergraduate students, Smedley and coworkers (6) investigated whether the 2D:4D digit ratio is predictive for depression severity and found that a more female (higher) 2D:4D digit ratio is correlated with more depressive symptoms in females only. Austin et al. (28) did not find a sex difference in the 2D:4D digit ratios of 79 male and 86 female students. Furthermore, males and females did not differ in depression ratings, while 2D:4D ratios were not associated with state depression, neither in males nor in females.

The heterogeneity of previous results is not surprising in light of the fact that sample sizes as well as effect sizes are mostly small in 2D:4D literature. Furthermore, the relation between 2D:4D ratio and depression has only been assessed in non-clinical samples. Therefore, further investigation of the associations between 2D:4D ratio and depression is clearly needed. We investigated the associations between 2D:4D ratios and major depression using a case–control design. Thus, we assessed the relation of 2D:4D ratios of both hands to depression by comparing 139 inpatients diagnosed with major depression according to DSM-IV criteria with 137 matched (for gender, handedness, and age as far as possible) healthy controls. In a first step, we compared 2D:4D ratios between patients with depression and control participants as a function of sex. In a second step, we related 2D:4D ratios to severity of depressive symptoms in both groups.

Basing on previous results, 1) we hypothesized that women report higher depressive symptom severity than do men (5). 2) We assumed that the 2D:4D ratio is positively correlated with depression severity in both depressed inpatients and controls with stronger associations for women than men based on the results of Smedley and colleagues (6). 3) We also hypothesized that patients suffering from depression have a higher 2D:4D ratio than have healthy controls based on the results of Smedley et al. (6). 4) Taking into account that the vast majority of previous studies examining 2D:4D ratios reported sex differences, we also expected sex differences when examining 2D:4D ratios with women showing higher 2D:4D ratios than men. 5) Last, we expected the correlations and group differences to be more pronounced in the right hand in line with the previous literature outlined above. Furthermore, we explored whether there is an interaction between group (depressed inpatients vs healthy controls) and sex in the prediction of 2D:4D ratios.

## Methods

### Participants

A total of 139 Caucasian patients with major depression, 90 women (64.7%) and 49 men, were recruited at the Clinic for Psychiatry and Psychotherapy III, University Hospital Ulm. All patients were diagnosed by resident psychiatrists according to Diagnostic and Statistical Manual of Mental Disorders (DSM-IV) (29) criteria based on a structured interview. Two patients were excluded from analyses with 2D:4D ratio because handscans were missing. All patients received stable antidepressant medication. Depression course (age of onset, number of depressive episodes, age of first inpatient treatment, number of inpatient treatments, and average duration of inpatient treatments) and dose equivalents of antidepressants (30) are presented in **Table 1**. Patients completed the BDI-II (31), a self-report questionnaire measuring depression severity by paper and pencil as well as an online in-house questionnaire containing a detailed assessment of medical history such as neurological and psychiatric disorders in addition to general medical status (number of participants in each of the BDI-II groups are presented in **Table 2**). Subthreshold depressive symptom severity in six patients may be explained by antidepressant treatment in the depression group.

**Table 1:** Descriptive statistics of depression history and dose equivalents for antidepressants for the depressive inpatients.

	n	Min	Max	Mean	SD
Age of onset	137	2	65	26.74	13.96
Number of depressive episodes	115	1	156	7.48	17.27
Age of first inpatient treatment	136	11	61	34.58	14.13
Number of inpatient treatments	136	1	80	2.96	6.97
Average duration of inpatient treatments	130	7.85	147.00	34.54	19.34
Dose equivalents AD	107	5.56	172.43	41.59	27.11

Age of onset in years; age of first inpatient treatment in years; average duration of inpatient treatment in days; dose equivalents AD: dose equivalent to 40 mg fluoxetine. Age of onset as well as number of depressive episodes was assessed according to patients’ self-report, with one patient reporting age 2 as age of onset and one patient reporting 156 depressive episodes. Since Hayasaka and colleagues (30) did not report dose equivalents for all available antidepressants, medication of n = 32 patients was excluded.

**Table 2:** Number and percentage of patients and control participants in the different BDI-II groups.

	Group			
BDI group	Depression		Control	
	n	%	n	%
No depression	6	4.3	137	100
Mild depression	17	12.2	0	0
Moderate depression	34	24.5	0	0
Severe depression	77	55.4	0	0

The six patients having subthreshold depressive symptom severity may be explained by antidepressant medication in the depression group.

For the control group, data sets of healthy Caucasian participants were drawn from the database of the Ulm Gene Brain Behavior Project (UGBBP). All healthy participants in the large group completed the BDI-II via online questionnaire without missing items as well as an online in-house questionnaire containing a detailed assessment of medical history such as neurological and psychiatric disorders in addition to general medical status. The control group comprised 137 healthy controls, 88 women (64.2%) and 49 men, without a history of psychiatric illness according to self-report and with a BDI-II score ≤ 13, which is below the cutoff for mild depression (31).

The depressed group and the control group were matched by sex, handedness, and age as far as possible (descriptive statistics are presented in **Table 3**). Handedness was assessed via self-report. Each participant had to decide between three options: right-handed, left-handed, and ambidextrous. Three participants were ambidextrous in the patient as well as in the control group. The number of left-handed participants in both groups was small and comparable (depressed, 11; healthy, 11). Four patients did not report their handedness and were therefore excluded from all analyses with 2D:4D ratio. We also filled out the Newcastle-Ottawa Quality Assessment Scale (NOS) for Case–Control Studies (32) (see the **Supplement**). All participants signed an informed written consent. Procedures of the study have been approved by the ethical review board of Ulm University.

**Table 3:** Descriptive statistics of the examined variables for both groups separately.

	n	Min	Max	Mean	SD
Age					
Depression	137	18	65	39.40	14.38
Control	137	18	63	28.74	9.28
BDI-II					
Depression	134	9	59	31.24	11.44
Control	137	0	12	4.95	3.49
Left 2D:4D					
Depression	115	0.8797	1.0900	0.9781	0.0358
Control	127	0.9267	1.0840	0.9809	0.0298
Right 2D:4D					
Depression	115	0.8808	1.0666	0.9769	0.0309
Control	131	0.9226	1.1110	0.9766	0.0298
Difference 2D:4D (L − R)					
Depression	103	0.0767	0.0551	0.0016	0.0231
Control	126	-0.0461	0.0525	0.0041	0.0209

The differences in sample size reflect patients not completing the BDI-II and handscans being non-analyzable.

The two groups differed significantly in age, with the depressed participants being significantly older than the healthy control group [*F*(1,272) = 53.17, *p* < .001]. The reason for the difficulty of matching age between both groups was that mean age in the large group comprising the UGBBP was much lower than mean age in the group of depressed inpatients. Age correlated significantly negatively with depression severity in the whole sample (*r* = −.24, *p* <.001) and in the group of depressive inpatients (*r* = −.22, *p* = .01) but not in the group of matched controls (*r* = −.08, *p* = .37). The 2D:4D ratio, however, is considered an age-independent trait (33, 34), which was substantiated by a control analysis resulting in non-significant correlation coefficients (Pearson, two-tailed) between age and 2D:4D ratio in the depression (left hand, *r* = .07, *p* = .44; right hand, *r* = .07, *p* = .48) as well as in the control group (left hand, *r* = .09, *p* = .30; right hand, *r* = .05, *p* = .54). Nonetheless, we used age as a covariate in all analyses performed.

### Questionnaires

The BDI-II (31) is a self-report questionnaire comprising 21 questions for the assessment of depression severity. The items are sadness, pessimism, feelings of failure, loss of pleasure, feelings of guilt, punishment feelings, self-dislike, self-criticalness, suicidal thoughts, crying, agitation, loss of interest, indecisiveness, worthlessness, loss of energy, change of sleeping habits, irritability, change of appetite, concentration difficulty, tiredness or fatigue, and loss of interest in sex. Cutoff scores differentiate between no depression (0–12), mild depression (13–19), moderate depression (20–29), and severe depression (30–63) (**Table 2**). Participants were instructed that the answers should reflect the previous 2 weeks.

### 2D:4D Ratio

A Canon Scanner (CanoScan LiDE110, Canon, Tokyo, Japan) with a resolution of 400 DPI was used for the scans of the right and left hands for the measurement of the length of ring fingers (4D) and index fingers (2D) of depressed patients and healthy participants. Two independent raters digitally measured the length from the middle of the basal crease to the tip of these fingers using GIMP2.8 Software (The GIMP Team, available on www.gimp.org). The on-screen resolution when measuring the finger lengths of 2D and 4D was 100%. The absolute finger length was measured in pixels. 2D:4D ratios were calculated for the right and left hands, resulting in two ratios for each hand due to two raters. Intraclass correlations between the two raters in the depressed sample were α = .95, 95% CI [.92;.96] for the right hand and α = .96, 95% CI [.95;.97] for the left hand. In the healthy control group, interrater correlations were α = .95, 95% CI [.92;.96] for the right hand and α = .96, 95% CI [.94;.97] for the left hand (all *p*-values <.001). As interrater reliability was high, mean ratios of the two raters were calculated for each hand, resulting in one 2D:4D ratio for each of the two hands of each patient and each healthy participant. Additionally, we analyzed the difference in mean 2D:4D ratio between the left hand and the right hand (L–R).

### Statistical Analyses

We conducted statistical analysis using R (35) and IBM SPSS Statistics (version 25, IBM, USA). Statistical significance was determined at *p* < .05 (two-sided test level). We first assessed whether there were significant sex differences in depression severity in depressed inpatients and in the healthy control group using a one-way ANCOVA, with gender as independent variable and age as covariate. Note that even though BDI-II scores were non-normally distributed according to the Shapiro–Wilk test in the group of matched controls (*Shapiro–Wilk* = 0.94, *df* = 137, *p* <.001), visual inspection of the boxplot and Q–Q plot did reveal neither the presence of outliers nor a severe deviation from normal distribution. Moreover, analyses of variance are not considered to be sensitive to moderate deviations from normality. Simulation studies showed that using a variety of non-normal distributions did not affect the rate of false-positive results (36–38). Furthermore, variances were homogenous across groups [studentized Breusch–Pagan test: inpatients, *BP*(4) = 4.59, *p* = .33; controls, *BP*(4) = 1.38, *p* = .85]. We therefore decided to use parametric tests. In order to assess whether depression severity was associated with the 2D:4D ratios, we calculated partial Pearson correlation coefficients between BDI-II score and 2D:4D ratios of both hands, with age as covariate. Note that since partial Spearman’s rank correlations provided similar results, we decided to report the results of the analyses with partial Pearson correlation coefficients. We analyzed the whole sample as well as the two experimental groups and sexes separately to see whether the association between BDI-II score and 2D:4D ratio differed between group and sex.

Moreover, we performed three 2 × 2 two-way ANCOVAs to assess the effects of group and sex on 2D:4D ratio. We conducted one ANCOVA each for the difference in 2D:4D ratio (L − R) and 2D:4D ratios of the right and left hands as dependent variables. Group and sex as well as their interaction term were entered as predictors. Age was used as covariate. The model therefore was as follows: 2D:4D ratio *=* constant + age + sex + group + sex * group. According to visual inspection of the boxplots, there were no outliers. There was homogeneity of error variances [Levene’s test: Difference (L − R), *F*(3,224) = 0.46, *p* = .71; left hand, *F*(3,233) = 2.47, *p* = .06; right hand, *F*(3,237) = 2.54, *p* = .06]. Significant interaction terms were additionally analyzed with Bonferroni *post hoc* tests in order to reveal mean differences between conditions.

We additionally performed a 2 × 2 × 2 mixed ANCOVA to assess the effects of group, sex, and hand on 2D:4D ratio. 2D:4D ratio was the dependent variable. Group and sex as well as their interaction term were entered as between-subjects variables. Hand (left vs right) was entered as within-subjects variable. We also included age as covariate. According to visual inspection of the boxplots, there were no outliers. There was no homogeneity of error variances [Levene’s test: *F*(3,224) = 2.84, *p* = .04]. There also was no homogeneity of covariances, as assessed by Box’s test (*p* = .03). Thus, results of this analysis have to be interpreted with caution because the prerequisites of an ANCOVA were not met. Nevertheless, this analysis can be found in the **Supplementary Material**. Bonferroni *post hoc* tests can still be interpreted and match the results of the aforementioned two-way ANCOVAs.

## Results

### Sex Differences in Depression Severity

The one-way ANCOVA for the healthy control group revealed no significant sex difference in BDI-II scores [male, *M* = 4.47, *SD* = 3.52; female, *M* = 5.22, *SD* = 3.46; *F*(1,134) = 1.05, *p* = .31].

In contrast, we found a significant sex difference regarding depression severity in the group of depressed inpatients [male, *M* = 27.34, *SD* = 11.13; female, *M* = 33.25, *SD* = 11.03; *F*(1,130) = 7.76, *p* = .006; *ƞ* *_p_* ^2^ = .061]. Women reported more depressive symptoms than men.

### Partial Correlations Between 2D:4D and Depression Severity

Contrary to our expectations, there was no significant association between BDI-II score and 2D:4D ratios, neither for the difference in 2D:4D ratio (L − R) nor for the absolute ratios of the left or right hands (**Table 4**). There were also no significant associations between BDI-II score and 2D:4D ratios when looking at groups, sexes, or the right-handed participants (the number of left-handed individuals only was *n* = 22) separately. Partial correlation coefficients ranged from *r* = −.15 to *r* = .18 (all *p*-values >.10, two-sided testing). Subgroup analyses of BDI-II groups (no depression, moderate depression, and severe depression; the group suffering from mild depressive symptom severity was omitted due to the small number of individuals comprising this group) did also not yield any significant associations (correlation coefficients ranged from *r* = −.14 to *r* = .18; all *p*-values >.10) (for scatterplots, see the Supplement). Inclusion of dose equivalents for antidepressants as covariate in the group of depressed inpatients did not yield any significant correlations between BDI-II scores and 2D:4D ratio (results not shown).

**Table 4:** Partial Pearson correlation coefficients between BDI-II scores and 2D:4D ratio with *p*-values.

BDI-II score	Left hand 2D:4D	Right hand 2D:4D	Difference 2D:4D (L − R)
Whole sample	−0.03 (.68)	−0.01 (.91)	−0.08 (.26)
Depression	0.08 (.41)	0.02 (.87)	0.04 (.70)
Control	−0.08 (.39)	0.03 (.76)	−0.12 (.20)
Men			
All	0.09 (.40)	0.13 (.23)	−0.06 (.61)
Depression	0.18 (.29)	0.08 (.67)	0.14 (.44)
Control	−0.12 (.43)	−0.10 (.52)	−0.04 (.80)
Women			
All	−0.12 (.16)	−0.07 (.43)	−0.10 (.25)
Depression	−0.01 (.97)	−0.02 (.86)	−0.01 (.91)
Control	−0.09 (.43)	−0.04 (.73)	−0.14 (.22)
Right-handed			
All	−0.00 (.99)	0.02 (.74)	−0.06 (.44)
Depression	0.07 (.48)	0.04 (.70)	0.03 (.81)
Control	−0.15 (.11)	−0.03 (.75)	−0.12 (.22)
Right-handed			
Men	0.05 (.67)	0.10 (.43)	−0.06 (.63)
Women	−0.05 (.58)	−0.01 (.95)	−0.07 (.42)

Covariate: age. p-values are in brackets (two-sided testing).

### Group and Sex Differences in 2D:4D Ratio

The two-way ANCOVA with the difference in 2D:4D ratio (L − R) as dependent variable revealed no significant main effects [group, *F*(1,223) = 2.24, *p* = .14, n.s.; sex, *F*(1,223) = 0.03, *p* = .87, n.s.]. The interaction term of group and sex had also no significant effect on the difference in 2D:4D ratio, *F*(1,223) = 1.94, *p* = .17 (n.s.).

**Figure 1:**
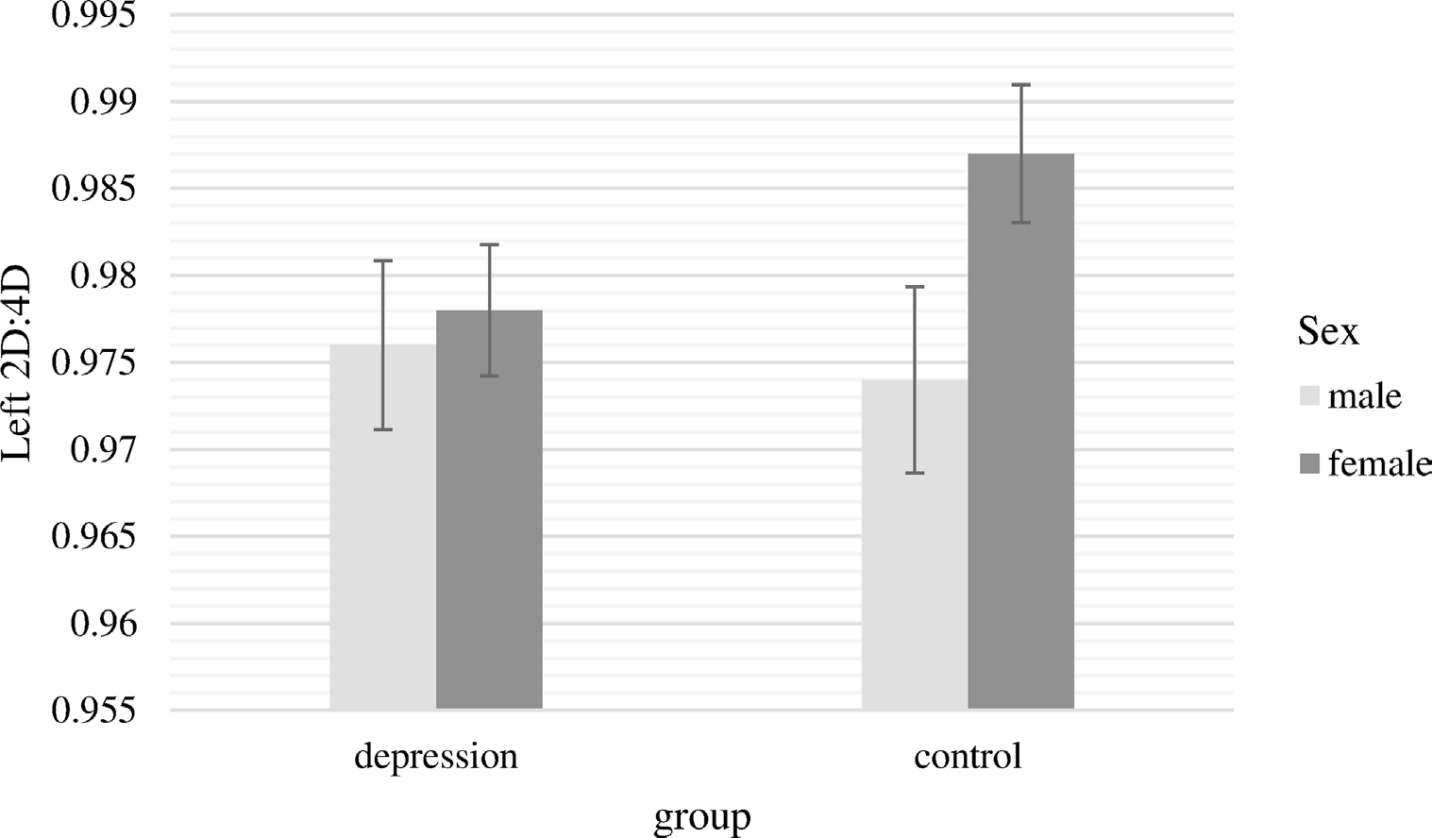
Mean 2D:4D ratio of the left hand with standard errors of the mean separately for group and sex (covariate: age).

The two-way ANCOVA with the 2D:4D ratio of the left hand as dependent variable revealed no significant main effects [group, *F*(1,232) = 0.46, *p* = .50, n.s.; sex, *F*(1,232) = 2.86, *p* = .09, n.s.]. The interaction term of group and sex had also no significant effect on 2D:4D ratio of the left hand, *F*(1,232) = 1.69, *p* = .20, n.s. (**Figure 1**).

**Figure 2:**
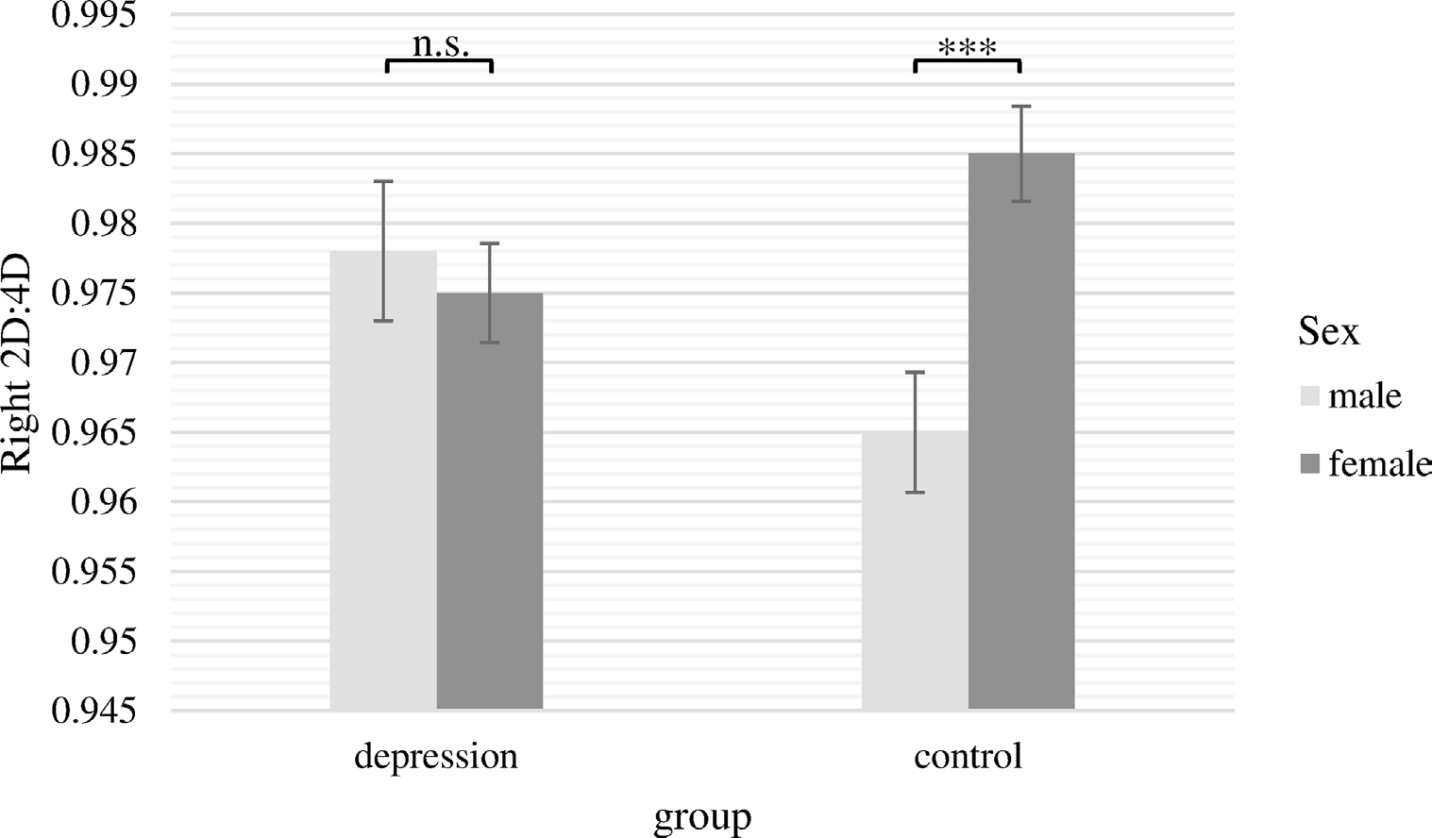
Mean 2D:4D ratio of the right hand with standard errors of the mean separately for group and sex (covariate: age). There were significant differences between males and females in the control group but not in the depression group. ***p < .001.

The two-way ANCOVA with the 2D:4D ratio of the right hand as dependent variable revealed a significant main effect of sex [*F*(1,236) = 4.28, *p* = .04, *ƞ* *_p_* ^2^ = .018]. In line with our assumptions, women had significantly higher 2D:4D ratios for the right hand. The main effect of group was not significant [*F*(1,236) = 0.10, *p* = .76]. Moreover, there was a significant interaction between group and sex [*F*(1,236) = 7.77, *p* = .01, *ƞ* *_p_* ^2^ = .032]. This interaction is presented in **Figure 2**. Bonferroni *post hoc* tests showed that 2D:4D ratio for women and men differed significantly only in the control group (*M* *_male_* = 0.965, *SE* *_male_* = 0.004; *M* *_female_* = 0.985, *SE* *_female_* = 0.003; *Difference* = −0.020, *p* <.001, *ƞ* *_p_* ^2^ = .052), whereas there was no significant sex difference in the depression group (*M* *_male_* = 0.978, *SE* *_male_* = 0.005; *M* *_female_* = 0.975, *SD* *_female_* = 0.004; *Difference* = 0.003, *p* = .64, n.s.). Within both sexes, 2D:4D ratio did not significantly differ between patients and controls, but there was a non-significant trend with males suffering from depression having a higher 2D:4D ratio than healthy men and females suffering from depression showing a lower 2D:4D ratio than healthy women (*Difference* *_male_* = 0.013;* p* = .06, n.s.; *Difference* *_female_* = 0.010; *p* = .06, n.s.).

## Discussion

The aim of our study was to assess whether 2D:4D finger ratios, reflecting prenatal testosterone exposure (39), differ between inpatients diagnosed with depression and healthy participants. 1) We hypothesized that women report higher depressive symptom severity than men (5). 2) We assumed that the 2D:4D ratio is positively correlated with depression severity in both depressed inpatients and controls, with stronger associations for women than men based on the results of Smedley and colleagues (6). 3) We also hypothesized that patients suffering from depression have a higher 2D:4D ratio than healthy controls based on the results of Smedley et al. (6). 4) Taking into account that the vast majority of previous studies examining 2D:4D ratios reported sex differences, we also expected sex differences when examining 2D:4D ratios with women showing higher 2D:4D ratios than do men. 5) Last, we expected the correlations and group differences to be more pronounced in the right hand in line with the previous literature outlined above. Furthermore, we explored whether there is an interaction between group (depressed inpatients vs healthy controls) and sex in the prediction of 2D:4D ratios.

We did not find a significant sex difference in depression severity in the healthy control group, which is not surprising due to the limited variance in BDI-II scores. However, female depressed inpatients showed significantly higher BDI-II scores when compared with male depressed inpatients. The non-significant sex difference in depression severity in healthy controls is in line with the results of Bailey and Hurd (26) reporting no significant sex difference in trait depression in a sample of undergraduate students. Moreover, Thayer and colleagues (40) found no significant sex difference in depressive symptom severity when examining individuals with low BDI-II scores (BDI-II score ≤ 6). However, when examining the group of individuals with relatively high depression severity (BDI-II score > 6), females showed significantly higher depression scores than males. According to Thayer and colleagues (40), healthy women exhibit greater emotional information processing due to high attention to emotions (41) paired with good emotional clarity and sufficient emotional repair strategies. In contrast, for depressed women, the high emotional attention combined with impaired anti-rumination strategies might lead to an emotional downward spiral and therefore higher depression severity (40).

Contrary to our expectation and some earlier work (6), in the present study, the 2D:4D ratio was not generally higher in patients with depression compared with healthy controls. Hence, there were no signs of a generally lower prenatal testosterone exposure in patients with depression. Instead, we found a significant interaction of group and sex for the 2D:4D ratio of the right hand. Significant sex differences in 2D:4D ratios were observed in healthy controls as expected, whereas in the group of depressed inpatients, sex differences in 2D:4D ratios were absent. This, in part, contrasts with the results of Bailey and Hurd (26) reporting that depression in men is associated with a higher more feminine 2D:4D digit ratio for the right hand. In our study, we found no significant difference in 2D:4D ratio between men in the depression and control groups. There was, however, a non-significant trend: depressive men tended to have a higher, more feminine 2D:4D digit ratio than have healthy men. Furthermore, we observed a non-significant difference, being lower in patients compared with controls, in the 2D:4D ratio within the group of women. These observations in combination with the significant interaction between depression diagnosis and sex indicate that depression is associated with the absence of sex differences in 2D:4D ratio of the right hand, i.e., 2D:4D ratio of the right hand is not an indicator of sex in patients suffering from depression. Our findings might be the result of prenatal levels of testosterone to estradiol influencing personality: high prenatal levels of testosterone relative to prenatal levels of estradiol are associated with lower 2D:4D ratios (39). Lower 2D:4D ratios, in turn, are associated with personality traits considered typically male such as aggressiveness (17) and a more masculine gender role identity. Conversely, higher 2D:4D ratios are related to a higher self-reported femininity (42). Thus, the absence of sex differences in 2D:4D ratios as found in our group of patients with depression might be associated with gender-atypical personality traits not matching societal gender stereotypes leading to a higher gender role conflict. Gender role conflict in turn is considered a predictor of depression and anxiety(43, 44). On the other hand, there are doubts concerning the robustness of within-sex correlations between 2D:4D ratios and personality traits considered typically male/female ( 45). The study by Manning and colleagues (42), however, has a large sample size and shows a consistent negative association between the 2D:4D ratio and self-reported masculinity.

The interaction of sex and group regarding 2D:4D ratio was significant only for the right hand (females had a higher 2D:4D ratio than men in the healthy control group but not in the group of depressed inpatients). This is in line with previous research reporting that the sex difference in 2D:4D is more prominent in the right hand than in the left hand (14, 15). Moreover, the difference in 2D:4D ratio between the right and left hands has been shown to be higher in right-handed than in left-handed participants (46). Like the vast majority of previous studies, our sample comprised mostly right-handed participants tending to have greater sex differences in 2D:4D ratios especially in the right hand. Therefore, the observed differences between the right and left hands in the analysis of the 2D:4D ratios as a function of sex and group might be caused by the right-handed participants. Since the number of left-handed participants was low in both the patient and control groups, a separate analysis of the left-handed subgroup was not possible.

Contrary to our expectations, correlation analyses between 2D:4D ratios of both hands and depression severity did not reveal any significant associations, neither in the entire sample nor in subsamples of patients and controls or women and men. In light of the lacking linear relationship between 2D:4D ratio and depression severity and our previous assumptions, 2D:4D might not be a linear predictor of depression severity. Instead, there might be a certain threshold of gender role conflict, which has to be exceeded in order to predispose an individual to the development of depressive symptomatology. We presumed that the absence of sex differences in 2D:4D ratios of the right hand is associated with sex atypical levels of prenatal testosterone (39) and results in sex atypical personality traits (17) and thereby in a higher gender role conflict ( 42–44). Gender role conflict has been shown to be associated with depression (43, 44).

It should also be noted that previous reports of an association between depression severity and 2D:4D digit ratio were heterogeneous (6, 26, 27). These heterogeneous findings might be a result of sample composition. In the present study, 139 inpatients who were diagnosed with major depression by psychiatrists according to DSM-IV criteria as well as 137 healthy controls were analyzed regarding associations of BDI-II scores and 2D:4D digit ratio. The participants in the earlier studies did not involve inpatients with a clinical diagnosis of depression. Thus, the samples of the presented study and those of earlier studies are not comparable. Whereas we investigated the two extremes regarding depression severity (very low depression severity in the control group and highly depressive inpatients in the depression group), previous studies most likely had a sample comprising mainly participants with no or mild depression as indicated, e.g., by the BDI-II means and standard deviations reported in the study of Smedley and colleagues (6): *M* *_male_* = 6.12, *SD* *_male_* = 4.99; *M* *_female_* = 9.31, *SD* *_female_* = 7.12. Furthermore, as depression severity is certainly a state variable changing over time whereas 2D:4D digit ratio is a stable trait variable, the association between both measures is necessarily limited.

Compared to previously published studies (6, 26, 27), the present investigation is based on a case-control design including depressed patients diagnosed according to DSM-IV criteria and healthy controls who had to pass an extensive screening procedure to exclude a possibly undiagnosed depression. The depressed patient group and the control group were matched for sex and handedness. Although the depressed patients and the healthy controls differed significantly in age, a correction regarding the age difference is negligible since the 2D:4D ratio is a stable age-independent trait (33, 34). But to avoid any confounding influences, we controlled for age in all our analyses.

Even though we examined inpatients diagnosed with depression and a sample of healthy controls, there are some limitations that need to be considered when interpreting the results of the current study. First, a study with monozygotic and dizygotic adult twins showed that genetic and environmental effects both affect the 2D:4D ratio (47). Due to the study design, the separation between genetics and environment was not possible in the current investigation. In addition, in the present study, all patients were treated with antidepressant medication and psychotherapy, thereby reducing depressive symptom severity. Antidepressant treatment or psychotherapy could have reduced the correlation between 2D:4D ratios and depressive symptoms. This limitation of our study, however, cannot be avoided, since depressive and potentially suicidal patients have to be treated adequately. Furthermore, there were procedural differences between depressive inpatients and healthy controls. While controls completed an online questionnaire, control cases completed a paper–pencil version. We used a paper–pencil version for inpatients to enhance commitment in this group burdened by difficulties in concentration and decision making. However, usage of different methods of measurement could have a confounding effect, even though there are studies suggesting invariance across different measurement methods at least for some instruments (48, 49). Moreover, handedness was assessed via self-report without using a validated instrument. There are, however, doubts that the usage of handedness scales yields reliable results (50). Last, we did not consider personality or gender role, variables potentially mediating the association between 2D:4D ratios and depression.

In conclusion, the present study is, to the best of our knowledge, the first to investigate 2D:4D ratios in inpatients with major depression using a case-control design. In patients with major depression, sex differences in the 2D:4D ratio of the right hand were absent, whereas in healthy controls, 2D:4D ratio was smaller in men than in women, as expected. 2D:4D ratios have previously been associated with masculinity/femininity traits (17, 42). We therefore speculate that the absence of sex differences in 2D:4D ratios of the right hand might result in an increased gender role conflict, because of the incongruence of one’s personality and the still prevailing but outdated societal definitions of masculinity and femininity. Gender role conflict might constitute a risk factor predisposing for depressive symptomatology. Future research should investigate 2D:4D ratios in a longitudinal design elucidating the predictive role of 2D:4D ratios for the development of personality traits and depression risk.

## Ethics Statement

This study was carried out in accordance with the recommendations of the Declaration of Helsinki, with written informed consent from all subjects. All subjects gave written informed consent in accordance with the Declaration of Helsinki. The protocol was approved by the Ethics Committee of Ulm University.

## Author Contributions

MK and CM conceived the study. KW-M and SS collected the data of the patients. JW and CS collected the data of the control participants and measured the finger length of all participants. SS and KW-M analyzed the data and wrote the first draft of the manuscript. All authors revised the manuscript and approved the final version.

## Funding

The position of CM is funded by a Heisenberg grant awarded to him by the German Research Foundation (MO 2363/3-2). JW receives stipend from the German Academic Scholarship Foundation (Studienstiftung des deutschen Volkes). CS receives stipend from the German Academic Scholarship Foundation (Studienstiftung des deutschen Volkes).

## Conflict of Interest Statement

The authors declare that the research was conducted in the absence of any commercial or financial relationships that could be construed as a potential conflict of interest.
