# Supplementary material for: Depression Is Associated With the Absence of Sex Differences in the 2D:4D Ratio of the Right Hand
Source: Front Psychiatry. 2019 Jul 16;10:483. doi: 10.3389/fpsyt.2019.00483 (PMC6660250; doi:10.3389/fpsyt.2019.00483)
Supplement: Supplementary file 1 [file DataSheet_1.docx]

Depression is associated with the absence of sex differences in the 2D:4D ratio of the right hand

**^1§^Simon Sanwald (M. Sc.), ^1§^Katharina Widenhorn-Müller* (PhD), ^2^Jennifer Wernicke (M. Sc.), ^2^Cornelia Sindermann (M. Sc.), ^1§^Markus Kiefer (PhD) and ^2§^Christian Montag (PhD)**

^1^Department of Psychiatry, Ulm University, Ulm, Germany

^2^Department of Molecular Psychology, Institute of Psychology and Education, Ulm University, Ulm, Germany

§ Both authors contributed equally to this work

***Correspondence:**Katharina Widenhorn-Müller

[Katharina.Widenhorn-mueller@uni-ulm.de](mailto:Katharina.Widenhorn-mueller@uni-ulm.de)

**Supplement:**

1. **Quality assessment for case control studies**

**NEWCASTLE - OTTAWA QUALITY ASSESSMENT SCALE**

**CASE-CONTROL STUDIES**

Note: A study can be awarded a maximum of one star (*) for each numbered item within the Selection and Exposure categories. A maximum of two stars can be given for Comparability.

Selection

1. Is the case definition adequate?
   1. **yes, with independent validation ***
      - **patients were diagnosed by psychiatrists of the Clinic for Psychiatry and Psychotherapy III before being considered participating in our study.**
   2. yes, e.g., record linkage or based on self reports
   3. no description
2. Representativeness of the cases
   1. consecutive or obviously representative series of cases *
   2. **potential for selection biases or not stated**
      - **all patients diagnosed with Major Depression without comorbid psychiatric disorder were asked to participate, but we cannot safely exclude that there are no biases.**
3. Selection of Controls
   1. **community controls ***
   2. hospital controls
   3. no description
4. Definition of Controls
   1. **no history of disease (endpoint) ***
   2. no description of source
5. Comparability
   1. Comparability of cases and controls on the basis of the design or analysis
      1. **study controls for _handedness_ (Select the most important factor.) ***
      2. **study controls for any additional factor * (This criteria could be modified to indicate specific control for a second important factor.)**
      - **age**
6. Exposure
   1. Ascertainment of exposure
      1. **secure record (eg surgical records) ***
      - **2D:4D ratio**
      1. structured interview where blind to case/control status *
      2. interview not blinded to case/control status
      3. **written self report or medical record only**
      - **but there was no treatment exposure**
      1. no description
   2. Same method of ascertainment for cases and controls
      1. **yes ***
      - **handscans were taken with the same scanner, the instruments were the same but controls filled out an online version of the questionnaires used**
      1. no
   3. Non-Response rate
      1. same rate for both groups *
      2. **non respondents described**
      - **non respondents only in the depression group due to the paper pencil version of the questionnaires.**
      1. rate different and no designation
7. **Scatterplots of the subgroup correlation analyses for BDI-II groups.**

| **** |  | |
| --- | --- | --- |
|  | |  |

Figure 1. Scatterplots for the association between depression severity and 2D:4D ratio of the right hand separately for each BDI-II group. The group suffering from mild depression was omitted due to lack of sample size. Partial Pearson correlation coefficients with covariate age: No depression: r_p_ = .03, n = 133, p = .71; moderate depression: r_p_ = .16, n = 25, p = .44; severe depression: r_p_ = .06, n = 58, p = .68.

|  |  |
| --- | --- |
|  |  |

Figure 2. Scatterplots for the association between depression severity and 2D:4D ratio of the left hand separately for each BDI-II group. The group suffering from mild depression was omitted due to lack of sample size. Partial Pearson correlation coefficients with covariate age: No depression: r_p_ = -.12, n = 129, p = .18; moderate depression: r_p_ = .18, n = 24, p = .38; severe depression: r_p_ = .02, n = 59, p = .91.

|  |  |
| --- | --- |
|  |  |

Figure 3. Scatterplots for the association between depression severity and the difference in 2D:4D ratio (L-R) separately for each BDI-II group. The group suffering from mild depression was omitted due to lack of sample size. Partial Pearson correlation coefficients with covariate age: No depression: r_p_ = -.14, n = 127, p = .10; moderate depression: r_p_ = -.05, n = 23, p = .81; severe depression: r_p_ = -.04, n = 55, p = .75.

1. **Mixed ANCOVA**

*Within subjects effects:*

| Variable | *F*(1,223) | *p* |
| --- | --- | --- |
| hand | 0.08 | .78 |
| hand*age | 0.99 | .32 |
| hand*sex | 0.03 | .87 |
| hand*group | 2.24 | .14 |
| hand*sex*group | 1.94 | .17 |

*Between subjects effects:*

| Variable | *F*(1,223) | *p* | *ƞ_p_^2^* |
| --- | --- | --- | --- |
| age | 3.64 | .06 | .016 |
| sex | 4.04 | .05 | .018 |
| group | 0.53 | .47 | .002 |
| sex*group | 4.27 | .04 | .019 |

*Post-hoc Bonferroni:*

In the control group there was a significant sex difference in mean 2D:4D ratio (males: *M* = 0.9730; *SD* = 0.0620; females: *M* = 0.9857; *SD* = 0.0309; post-hoc *Bonferroni*: *p* = .002).

In the group of depressed inpatients there was no significant sex difference in mean 2D:4D ratio (males: *M* = 0.9771; *SD* = 0.0419; females: *M* = 0.9781; *SD* = 0.0329; post-hoc *Bonferroni*: *p* = .98).

There was also a significant difference in mean 2D:4D ratio between females suffering from depression and females in the healthy control group (post-hoc *Bonferroni*: *p* = .03) but no difference in mean 2D:4D ratio comparing men from the depression to men from the control group (post-hoc *Bonferroni*: *p* = .44).
